# Body composition in premanifest Huntington’s disease reveals lower bone density compared to controls.

**DOI:** 10.1371/currents.RRN1214

**Published:** 2011-04-11

**Authors:** Anna O. G. Goodman, Roger A Barker

**Affiliations:** University of Cambridge

## Abstract

Huntington’s disease (HD) is a debilitating autosomal dominant, neurodegenerative disease with a fatal prognosis. Classical features include motor disturbances, dementia and psychiatric problems but are not restricted to this triad as patients often experience other abnormalities such as unintended weight loss, the exact cause of which is unknown.

We studied the body composition of 25 premanifest HD and compared it to 25 control subjects using a dual energy x-ray absorptiometer (DEXA) scan. Like the R6/2 transgenic mouse model, we identified significantly lower bone mineral density z-scores in premanifest individuals, that was not related to any difference in testosterone, cortisol, leptin or Vitamin D levels.

These results identify an early gene-related change that occurs in HD which not only could lead to a potential biomarker for the disease, but given it is also seen in other manifest neurodegenerative diseases, could also reveal a common disease related process.

## Introduction

Huntington’s disease (HD) is a fatal autosomal dominant neurodegenerative condition that affects approximately 4 to 8 individuals per 100,000[1] and typically presents between 35 and 45 years of age.  The disease runs a debilitating and progressive course with an average survival of 10-25 years after disease onset.[2]  It is caused by an abnormal cytosine-adenosine-guanosine (CAG) expansion[3] in exon 1 of the huntingtin gene[4] leading to the expression of mutant huntingtin (htt)[5] [6] which is expressed ubiquitously throughout the brain and body.[7] [8]  Patients classically develop involuntary movements including chorea, a progressive dementia[9] [10] and psychiatric disturbances.[11]  However a number of other features including sleep abnormalities, circadian rhythm changes, increased appetite and metabolic alterations and weight loss are also common in the disease and can often precede motor abnormalities.[12] [13] [14] [15] [16]

In the R6/2 transgenic mouse[17], mutant huntingtin (htt) expression is not only seen in the brain, but also in a range of peripheral tissues.[18] [19] [7] [20]  Involuntary movements, impaired cognitive performance and epileptic seizures have all been seen in these mice[17] and are also features of the human condition.[21] [22]  In addition to expressing similar classical features to the human disease, R6/2 mice also lose weight and experience muscle atrophy, abdominal fat accumulation and reduced bone mineral density, which interestingly has been found to actually precede the weight loss.[23] [24]

As part of an ongoing study on metabolic abnormalities in HD, we have been investigating a group of premanifest HD individuals over time using a number of different parameters.  In this paper we report on the change in body composition in such individuals and surprising show that premanifest individuals have significantly lower bone density compared to controls, that does not have an obvious explanation as they have normal levels of testosterone, cortisol, leptin and Vitamin D compared to age and sex matched controls.

## Materials and Methods

### Subjects

#### Patients

25 premanifest HD were selected according to the following criteria: (1) CAG repeat length > 39; (2) no known metabolic, endocrine or sleep disorder; (3) on no medication known to affect metabolism and/or endocrine function; (4) No motor features of HD as defined by a score of < 3 on the diagnostic confidence on the Unified Huntington’s disease Rating Scale (UHDRS).

#### Control subjects

25 sex, age and ethnicity matched control subjects were recruited via general advertisement and were included only if they had (1) no known metabolic, endocrine or sleep disorder and (2) on no medication known to affect metabolism and/or endocrine function.

**

#### Whole body composition

Whole body composition was measured using the Lunar Prodigy dual energy x-ray absorptiometry (DEXA; GE Lunar Corp., Madison, WI).  The DEXA scan builds up a whole body image using low intensity x-ray filtered into two energy spectra.  Fat and lean tissues are differentiated by differences in their absorption of x-ray at the two energies.  Where bone is present, discrimination is between bone and soft tissue, and generates a measure of bone mineral content.  

#### Blood samples

Blood samples for testosterone, cortisol, Vitamin D and leptin were taken at 8am, before the participant got out of bed and after an 8 hour fast.

#### Analysis

Two scores were generated using the DEXA associated software:  

*T score*; the amount of bone the participant has in relation to a young adult of the same gender with peak bone mass. A score above -1 is normal. A score between -1 and -2.5 is classified as osteopenia, the first stage of bone loss. A score below -2.5 is defined as osteoporosis. 

*Z score* ; the amount of bone the participant has in comparison to other people of their age group, gender, and size. A Student’s *t*-test was carried out between study groups to show any differences between the groups.

Testosterone levels of 0.5-3.0 *nmol*/*L* (females) and 8-29 *nmol/L* (males) were considered to be within the normal range.  

Cortisol levels of 280-650 *nmol/L* (males and females) were considered to be within the normal range.

Vitamin D (Total 25 OH (D2+D3)) levels > 50 *nmol/L *(males and females) were considered to be within the normal range.  30-50 *nmol/L *Vitamin D was considered suboptimal and treatment may be required if clinically indicated.  15-30 *nmol/L* represents a moderate deficiency, treatment usually required.  <15 *nmol/L*: represents a severe deficiency, treatment required.

Leptin levels were considered to be normal if they fell within the given ranges (Department of Clinical Biochemistry, Addenbrooke’s Hospital, Cambridge):

#### Men

BMI <25:  (0.1 - 22.8 *ng/ml*); BMI 25-30: (0.5 – 26.3 *ng/ml*); BMI 30-35 (2.1 – 36.7 *ng/ml*); BMI >35 (7.8 – 31.7 *ng/ml*)

#### Women

BMI <25:  (0.2 – 45.8 *ng/ml*); BMI 25-30: (3.0 – 65.7 *ng/ml*); BMI 30-35 (8.1 – 79.1 *ng/ml*); BMI >35 (11.9 – 137.4 *ng/ml*)

A significance level of *p* < 0.05 was used for all statistical tests performed.

## Results

**Figure 1.**
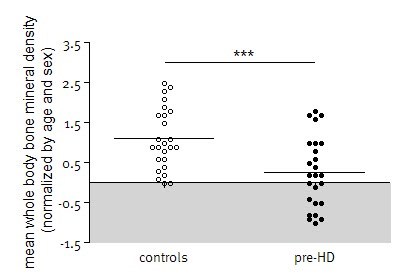
Vertical scatterplot of the bone mineral density *z*-scores in pre-HD individuals compared to controls.

BMD *z*-scores of the premanifest individuals and controls showed a significantly lower mean score in the premanifest group (mean patient *z* score = 0.27 vs. control *z*-score = 1.11, *p* < 0.001), see Table 1 and Figure 1.  When the sexes were compared independently, significant differences were found in each gender although the mean differences in males was more significant than in females (males *p*<0.01, females *p*<0.05).  *z*-scores were not significantly correlated to BMI (*r* = 0.025, *p* = 0.86) or age (*r* = 0.045, *p* = 0.76).  The BMD T-scores were also significantly lower in the premanifest group (mean premanifest *T* score = 0.27 vs. control *z*-score = 0.89, *p* < 0.04).   

There was no significant difference between premanifest individuals and controls for cortisol, vitamin D, leptin and testosterone levels.  There was no significant correlation with BMD z-score and estimated number of years to disease onset.  The Pre-HD and control groups had the same proportion of individuals who were in full-time employment vs. those who were not working.

**Table:** 

** **	**Age**	**Sex**	**BMI**	**Fat**		**BMD**		**Testosterone**		**Cortisol**	**Vitamin D**	**Leptin**
* *	*(years)*	*(m:f)*	* *	*%*	*z-score*	*T-score*	*z-score*	*nnmol/L*		*nnmol/L*	*nnmol/L*	*ng/ml*
* *	* *	* *	* *	* *	* *	* *	* *	*m*	*f*	* *	* *	* *
Controls	46.7 ± 13.3 (24.8 – 71.6)	10:15	24.7 ± 2.7 (21.1 – 31.7)	31.6 ± 7.9 (15.9 – 46.7)	0.68 ± 1.1 (-1.1 – 3.2)	0.89 ± 1.0 (-0.7 – 2.5)	1.11 ± 0.8 (0.0 – 2.5)	18.6 ± 6.3 (6.9 – 26.1)	1.51 ± 0.6) (0.1 – 2.3)	351.8 ± 89.3 (259 – 503)	66.0 ± 21.4 (8.5 – 196.8)	10.3 ± 7.5 (2.5 – 25.6)
												
Pre-HD	46.0 ± 12.4 (22.4 – 67.5)	10:15	26.3 ± 5.2 (21.1 – 40.7)	33.8 ± 10.9 (14.4 – 52.1)	0.45 ± 1.2 (-1.5 – 3.4)	0.27 ± 1.1 (-2.4 – 2.0)	0.27 ± 0.9 (-1.0 – 1.8)	17.43 ± 8.2 (8.7 ± 33.1)	1.4 ± 0.3 (0.9 – 1.9)	366.1 ± 69.8 (209 – 519)	64.5 ± 39.3 (19.5 – 99.5)	20.3 ± 30.6 (0.4 – 125)
												
Significance level	0.70	.	0.16	0.40	0.50	0.04	0.001	0.73	0.68	0.56	0.87	0.15

Abbreviations: BMD, bone mineral density; BMI, body mass index; cm^2^, centimeter squared; f, female; g, grams; HD, Huntington’s disease; ; L, litre; m; male; ml, milliliters; ng, nanograms; nnmol, nanomoles per liter.

*** *p*<0.001

****

## Discussion

Weight loss commonly occurs in HD and follows a progressive course[25] that begins in premanifest individuals[26] and ends with advanced stage patients experiencing profound cachexia.[27]  In this study, we carried out whole-body composition by means of a DEXA scanner in premanifest individuals and controls to see whether any differences could be found between groups in terms of fat, lean soft tissue and bone density content at a stage ahead of obvious disease manifestation.  

We identified significantly lower bone mineral density levels (*z*-scores) in premanifest individuals compared to controls, which suggest that such patients could be at greater risk of fracture.  This significance was greater in males premanifest individuals than in the females (males* p*<0.01, females *p*<0.05).  

The exact cause of this abnormality is unknown although there are various possible suggestions.  Low levels of testosterone have been shown to have a detrimental effect on bone density in men[28] and indeed significantly lower levels of testosterone have been identified in HD.[29]   However, we did not find a significant difference in testosterone levels between groups.  Glucocorticoids induce osteoblast apoptosis and increase osteoclast survival and activity and indeed elevated levels have been found in HD.[23]  However, in this study we did not find a significant difference in cortisol levels between groups.  Vitamin D insufficiency has also been linked to poor bone density,[30] however in our study our groups did not significantly differ.  Altered leptin levels have been found in both HD patients[31] and transgenic mice.[32]  One of leptin’s functions is to affect bone mass[33] [34] by inducing bone loss,[35] yet again we found no significant difference in leptin levels between study groups.  

 Accelerated bone loss has been identified in other neurodegenerative disorders such as Alzheimer’s disease (AD) and Parkinson’s disease (PD).  A recent study investigating BMD in early AD and its relationship to brain structure and cognition has shown that BMD is reduced in the earliest clinical stages of AD and is associated with brain atrophy and memory decline, suggesting that central mechanisms may contribute to bone loss in early AD.  Interestingly, low BMD was associated particularly with low volume in the hypothalamus,[36] suggesting that central mechanisms of bone remodeling may be disrupted by neurodegeneration.[37]  In HD, atrophy has been identified in a range of brain regions including the hypothalamus[38] [39] and thus this could be an important factor, although volumetric brain imaging would need to be done to prove this and was not part of this study.

There are certain limitations to our study.  Our control group appears to have a higher mean than the DEXA reference data.  Although our controls were randomly recruited and were age, sex and ethnicity matched, a larger sample size would be a better representation of the general population.  In addition, although the Pre-HD and control groups had the same proportion of individuals in full-time employment vs. those who were not working, since exercise can affect bone quality, it would have been useful to have obtained a measure of free-living activity, both in terms frequency and type of exercise, to see whether there was a relationship between activity and bone density in these groups.

In conclusion, in this study we used DEXA imaging to identify a significantly lower bone density in premanifest individuals compared to controls.  The cause of this finding is not known at this time but is not related to differences in testosterone, cortisol, leptin or Vitamin D.  These results suggest that early gene-related changes may be occurring in the bone which could not only identify potential biomarkers for the disease, but its similarity with other neurodegenerative diseases could reveal a common disease related process.  At the very least, this study highlights an area of vulnerability in patients that clinicians may be advised to monitor in order to reduce fractures should it be proven that the early abnormalities reported in this paper persist or worsen in manifest disease.

## Acknowledgments

We wish to thank all of our participants for their time and help, as well as all the staff at the WTCRF, Addenbrooke's Hospital who helped with this study.  We would also like to thank the National Institute for Health Research Cambridge Biomedical Research Centre Core Biochemistry Assay Laboratory for the analysis of Leptin and Amy Munro and the Biochemistry team at Addenbrooke’s Hospital for their help with the analysis of the testosterone, cortisol and Vitamin D.  We would also like to thank Professor Juliet Compston for her expert help and advice.  

## Author roles

*Dr. Anna Goodman*  Conception, organization and execution of study.  Design, execution and review of statistical analysis.  Writing of the first draft and review of manuscript.

*Dr. Roger Barker*  Conception and organization of study.  Design and review and critique of statistical analysis.  Review and critique of manuscript.

## Conflicts of Interest

Both authors have declared that they have no potential conflicts of interest related to this manuscript.  

## Financial Disclosure

All work carried out in this manuscript was paid for by the Cure Huntington’s Disease Initiative (CHDI).

## Ethics Approval

All research carried out was given full ethical approval from the Cambridge Research Ethics Committee.
